# Clinical Assessment of Prostate Displacement and Planning Target Volume Margins for Stereotactic Body Radiotherapy of Prostate Cancer

**DOI:** 10.3389/fonc.2020.00539

**Published:** 2020-04-16

**Authors:** Rebecca Levin-Epstein, George Qiao-Guan, Jesus E. Juarez, Zhouhuizi Shen, Michael L. Steinberg, Dan Ruan, Luca Valle, Nicholas G. Nickols, Patrick A. Kupelian, Christopher R. King, Minsong Cao, Amar U. Kishan

**Affiliations:** ^1^Department of Radiation Oncology, University of California, Los Angeles, Los Angeles, CA, United States; ^2^Case Western Reserve School of Medicine, Cleveland, OH, United States; ^3^Department of Radiation Oncology, VA Greater Los Angeles Healthcare System, Los Angeles, CA, United States; ^4^Department of Urology, University of California, Los Angeles, Los Angeles, CA, United States

**Keywords:** prostate cancer, stereotactic body radiation therapy, SBRT, prostate motion, planning target volume, margins, image-guidance

## Abstract

**Purpose:** To assess the optimal planning target volume (PTV) margins for stereotactic body radiotherapy (SBRT) of prostate cancer based on inter- and intra-fractional prostate motion determined from daily image guidance.

**Methods and Materials:** Two hundred and five patients who were enrolled on two prospective studies of SBRT (8 Gy × 5 fractions) for localized prostate cancer treated at a single institution between 2012 and 2017 had complete inter- and intra-fractional shift data available. All patients had scheduled kilovoltage planar imaging during SBRT with rigid registration to intraprostatic fiducials prior to each of four half-arcs delivered per fraction, as well as cone beam CT verification of anatomy prior to each fraction. Inter- and intra- fractional shift data were obtained to estimate the required PTV margins based on the classic van Herk formula. Inter- and intra-fractional motion were compared between patients with and without severe toxicities using the independent two-sample Wilcoxon test.

**Results:** The margins required to account for inter-fractional motion were estimated to be 0.99, 1.52, and 1.45 cm in lateral (LR), longitudinal (SI), and vertical (AP) directions, respectively. The margins required to account for intra-fractional motion were estimated to be 0.19, 0.27, and 0.31 cm in LR, SI and AP directions, respectively. Large intra-fractional shifts were mostly observed in the SI and AP directions, with 2.0 and 5.4% of patients experiencing average intra-fractional motion >3 mm in the SI and AP directions, respectively, compared with none experiencing mean shifts >3 mm in the LR direction. Six patients experienced grade 3 gastrointestinal or genitourinary toxicity. There were no significant differences in mean inter- or intra-fractional motion in any of the cardinal directions compared to patients without severe toxicity (inter-fractional *p* = 0.46–0.99, intra-fractional *p* = 0.10–0.84).

**Conclusion:** The inter- and intra-fractional margins estimated from this study are in line with prior reported values. Intra-fractional prostate motion was generally small with larger margins required for the SI and AP directions, notably just slightly exceeding the commonly used 3 mm posterior PTV margin even with realignment between half-arcs. Development of severe toxicity was not significantly associated with the degree of inter- or intra-fractional motion.

## Introduction

Extreme hypofractionation using stereotactic body radiotherapy (SBRT) for localized prostate cancer (PCa) is recognized as an appropriate treatment option with a favorable toxicity profile for men with localized low- to intermediate-risk PCa (1–5). Current utilization trends indicate an uptake in SBRT adoption (6, 7), which will likely be increased with dissemination of long-term safety data from the HYPO-RT-PC trial and acute toxicity information from the PACE-B trial (1, 5). By definition, SBRT necessitates a high level of accuracy and precision during treatment delivery, as each fraction is responsible for delivering a high proportion of the total prescription dose (2, 3). Thus, errors in the precision and accuracy of actual dose delivered can lead to incomplete target coverage and/or overdosing of adjacent organs-at-risk (8, 9).

Both systematic and random errors contribute to uncertainty in dose delivery. Systematic errors encompass errors in the plan preparation process, including acquisition of the planning scan, target delineation, and treatment planning, and occur upstream of treatment delivery. In treatment execution, both systematic, and random errors can occur, including those related to uncertainties in patient setup as well as target motion. These errors can manifest as both inter- and intra-fractional errors (10).

Image-guided inter-fractional setup is associated with improved accuracy of target localization, lower rates of toxicity, and improved biochemical control (11–13). Image-guidance using solely pre-treatment setup, however, is not sensitive to intra-fractional changes in target positioning during dose delivery, which can be significant (14). Variables affecting intra-fractional target motion include bladder and rectal filling, treatment time, target size, and tissue density (15–18). Rectal and bladder filling are generally the greatest contributors to prostate displacement, and as a result, prostate displacement tends to be greatest in the superior-inferior (SI) and anterior-posterior (AP) directions (9, 17–20). Motions can be transient, such as those created by movement of gas through the rectum, or sustained, such as continuous filling of the bladder and rectum (11).

Formulas to define the required margins for adequate target coverage have been developed, such as the classic van Herk formula (10, 21). Multiple image-guided motion management strategies exist (22), and a wide range of required prostate radiotherapy margins have been proposed based on varied techniques. However, the numbers of patients included in studies to estimate required margins are relatively modest, and proposed margins based on data from the intra-fractional, inter-arc kilovoltage (kV) imaging method is lacking (8, 9, 16, 19, 20, 23–27).

Since 2010, we have treated patients with PCa using gantry-mounted linear accelerator-based SBRT on two prospective studies. Our consistent motion management technique has involved inter-fractional and intra-fractional rigid registration to implanted prostatic fiducials using kV orthogonal imaging. Here, we present a detailed retrospective analysis of shift data from these patients, designed to guide the development of sufficient margins in the context of prostate SBRT. We also evaluate the association between shifts and severe toxicity.

## Materials and Methods

### Study Population

Our institution has routinely been delivering 40 Gy in five fractions using gantry-based SBRT since 2010 on prospective studies for both low- and intermediate-risk disease (NCT01059513) as well as high-risk disease (NCT02296229). The primary study population included 205 patients enrolled on either trial who had shift data available for analysis.

### Treatment Planning

For all patients, three gold fiducial markers were implanted transperineally under ultrasound guidance into the left base, right base, and apex of the prostate. Patients subsequently underwent a CT simulation scan obtained with 1.5 mm slice thickness. Patients were instructed to empty their bladder 30–60 min prior to scanning and then drink 16 ounces of water to attain a comfortably full bladder. Patients were encouraged to have an empty rectum; however, no specific bowel preparation was prescribed, apart from those who underwent same-day fiducial insertion and were required to perform an enema the night prior. No rectal immobilization or rectal spacer devices were used. The same bladder and bowel preparation instructions were given for each day of treatment. Patients were simulated in the supine position with a vac-loc cradle and knee wedge for comfort.

The clinical target volume (CTV) consisted of the prostate gland only in 188 (91.7%) patients. Treatment of the seminal vesicles and pelvic lymph nodes was performed in a small fraction of patients (*n* = 17, 8.3%) at the discretion of the treating physician, with nodal therapy (25 Gy over 5 fractions) allowed only for patients with high-risk disease. The prostate CTV was expanded 5 mm isotropically, with a reduction to 3–5 mm posteriorly, to create the planning target volume (PTV). The use of prostate MRI in the contouring process was variable, as this practice was emerging during the time period over which patients were treated. Treatment planning was performed using the Eclipse treatment planning system with RapidArc volumetric arc therapy (Varian Medical Systems, Palo Alto, CA). All patients were treated to 40 Gy over five fractions, delivered every other day via a gantry-mounted linear accelerator, either on the Novalis Tx (Varian Medical Systems, Inc., Palo Alto, CA) or TrueBeam (Varian Medical Systems, Inc., Palo Alto, CA). Treatment was delivered using four partial half arcs. Our institutional median (and interquartile range [IQR]) dose-volume parameters achieved for the PTV, rectum, and bladder are: PTV V_40Gy_ = 95% (optimized to 95%), rectal V_20Gy_ = 19.8% (15.6–25.4%), rectal V_36Gy_ = 2.9% (2.3–3.8%), rectal V_40Gy_ = 1.1% (0.6–1.5%), bladder V_20Gy_ = 14.5% (9.3–20.6%), and bladder V_40Gy_ = 6.9% (4.3–9.9%).

### Treatment Delivery and Margin Calculations

**Figure 1:**
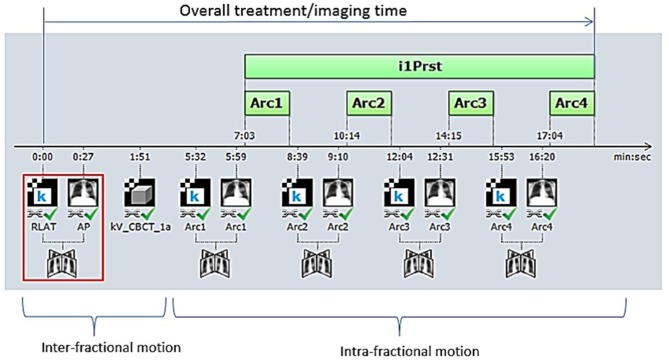
Our image-guided work flow during the treatment of stereotactic body radiation therapy for prostate cancer. Initially, patient positioning is accomplished by orthogonal image pair or stereoscopic X-ray imaging with ExacTrac® and cone-beam computed tomography (CBCT) before treatment beam-on. Repeat orthogonal image pair or stereoscopic X-ray imaging is obtained prior to each beam delivery (each of three remaining half-arcs) to account for intra-fractional motion. Each half-arc is delivered over ~2–3 min.

Initial daily alignment was conducted using either a three-point laser-guided setup to tattoos or the ExacTrac optical system for men treated on TrueBeam or NovalisTx, respectively. All patients underwent kV or MV planar imaging detecting implanted fiducial markers and cone beam CT (CBCT) verification of anatomy prior to treatment for inter-fraction motion correction. Planar image guidance with orthogonal kV x-ray images were subsequently obtained before each treatment field for intra-fraction motion correction (Figure 1). For patients treated on NovalisTx, ExacTrac planar imaging was used in place of kV or MV planar imaging. Our motion management strategy focused on rigid registration to the prostatic fiducials, even if pelvic nodal treatment was included. The projections of the implanted fiducials on the digital reconstructed radiograph was determined by identifying the locations of the fiducials in the reference CT during the planning phase. Fiducial marker match was performed by aligning the locations of the marker positions detected on the planar images to the calculated projections, and the resultant couch shift correction was calculated by the IGRT software provided with the treatment machine. Our institutional threshold for requisite implementation of shifts is a displacement of ≥1 mm.

Daily inter- and intra- fractional shift data were obtained to calculate the required PTV margins based on the classic van Herk formula, 2.5Σ + 0.7σ, in which Σ represents the standard deviation of the systematic error and σ represents the standard deviation of the random error (10, 21). For inter-fractional motion, mean and standard deviations of daily shifts in left-right (LR), superior-inferior (SI), and anterior-posterior (AP) directions were first obtained from kV image matching of fiducial seeds for each patient. The group systematic error was calculated as the average of all the individual means and Σ was then estimated by the standard deviation of all the patient means. The standard deviation of random error (σ) was determined by computing the root mean square of the standard deviation of each patient's shifts. To calculate intra-fractional motion, the absolute displacement in each of the three planes (LR, SI, and AP) was calculated for each image pair between half-arcs, and the average for each direction was obtained to determine mean displacement in the LR, SI, and AP directions for a given fraction. These mean displacements per fraction were then averaged to obtain the final mean displacement in the LR, SI, and AP directions for each patient. The standard deviation of the systematic (Σ) and random error (σ) were then calculated using the similar fashion as the inter-fraction motion.

Differences in prostate displacement over the sequential phases of treatment were also assessed. Prostate displacement in the three cardinal directions was measured during the initial IGRT and alignment phase, and for the individual partial arcs with corresponding subsequent intra-fractional imaging (arcs 1–3).

### Statistical Analysis

We identified six patients who experienced late grade ≥3 gastrointestinal (GI) or genitourinary toxicity (GU), by the Common Terminology for Adverse Events version 4.03. Inter- and intra-fractional motion was compared between patients with severe toxicities and the control group using the independent two-sample Wilcoxon test.

## Results

Baseline participant characteristics are presented in Table 1. Median age was 70 years old (range 44–87). Median prostate CTV was 58.7 cc (IQR 46.4–75.4 cc). The majority of patients had either cT1c or cT2a disease.

**Table 1:** Patient characteristics.

Total participants (n)	205
Median age, (range), in years	70 (44–87)
Median clinical target volume, (range), [interquartile range], in cubic centimeters (cc)	58.7 cc (46.4–75.4 cc) [23.8–154.3 cc]
Median initial PSA, (range)	6.9 ng/mL (0.05–70 ng/mL)
Distribution of T stage (%)	
cT1c	72.5%
cT2a	19.4%
cT2b	3.3%
cT2C	1.4%
cT3a	1.9%
cT3b	0.5%

### Treatment Time

The mean overall treatment time, encompassing inter- and intra-fractional imaging, alignment, and arc delivery, was 15.6 min (SD 8.1), with a median of 14.0 min (IQR 12.2–16.2). The mean time devoted to delivery of each partial arc, including the intra-fractional imaging, was 2.0 min (SD 0.4), with a median of 2.3 min (IQR 1.1–2.7). Treatment preparation, including inter-fractional imaging and alignment prior to beam-on, required an average 7.5 min (SD 3.9).

### Prostate Displacement and Required Margins

Mean prostate displacement in the LR, SI, and AP directions is shown in Table 2. Mean (± 1 standard deviation [SD]) inter-fractional target displacement in the LR, SI, and AP directions was −0.03 ± 0.30 cm, −0.06 ± 0.43 cm, and −0.06 ± 0.45 cm, respectively. Mean (± 1 SD) intra-fractional target displacement in the LR, SI, and AP directions was 0.07 ± 0.05 cm, 0.13 ± 0.07 cm, and 0.14 ± 0.09 cm, respectively.

**Table 2:** Mean, standard deviation (SD, Σ), and root mean square (RMS, σ) of inter- and intra- fractional motion of the prostate for patients treated with 5-fraction stereotactic body radiation therapy.

	**Inter-fractional**			**Intra-fractional**		
	**LR**	**SI**	**AP**	**LR**	**SI**	**AP**
**Mean (M)**	−0.03	−0.06	−0.06	0.07	0.13	0.14
**SD (Σ)**	0.30	0.43	0.45	0.05	0.07	0.09
**RMS (σ)**	0.35	0.63	0.47	0.09	0.12	0.13
**Margin (cm)**	0.99	1.52	1.45	0.19	0.27	0.31

*Calculated inter- and intra-fractional planning target volume margins required for prostate stereotactic body radiation therapy are presented. Inter-fractional margins reflect expansions that would be required if no inter-fractional image guidance were employed*.

**Figure 2:**
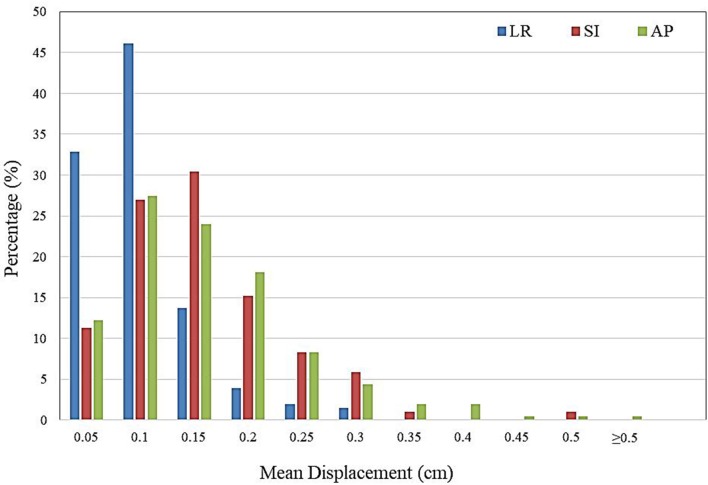
Histogram demonstrating frequency of mean intra-fractional target displacement in the lateral (LR), longitudinal (SI), and vertical (AP) directions for all patients, as measured by three sets of intra-fractional orthogonal imaging.

The distribution of intra-fractional target motion in the LR, SI, and AP directions is shown in Figure 2. Mean displacement was greatest in the AP direction, with 5.4% of patients experiencing mean AP displacement >3 mm and 0.5% of patients experiencing mean AP displacement of >5 mm. In the SI direction, 2.0% of patients experienced mean displacement >3 mm, and none experienced mean >5 mm SI displacement. No patients experienced mean LR displacement >3 mm.

The inter-fractional margins, which represent the margins that would need to be implemented if alignment were purely based on three-point setup or the optical system, were estimated to be 0.99, 1.52, and 1.45 cm in LR, SI, and AP directions, respectively. With implementation of image-guided setup, the required margins to account for intra-fractional motion are 0.19, 0.27, and 0.31 cm in LR, SI and AP directions, respectively (Table 2).

### Prostate Displacement Across Phases of Treatment

**Figure 3:**
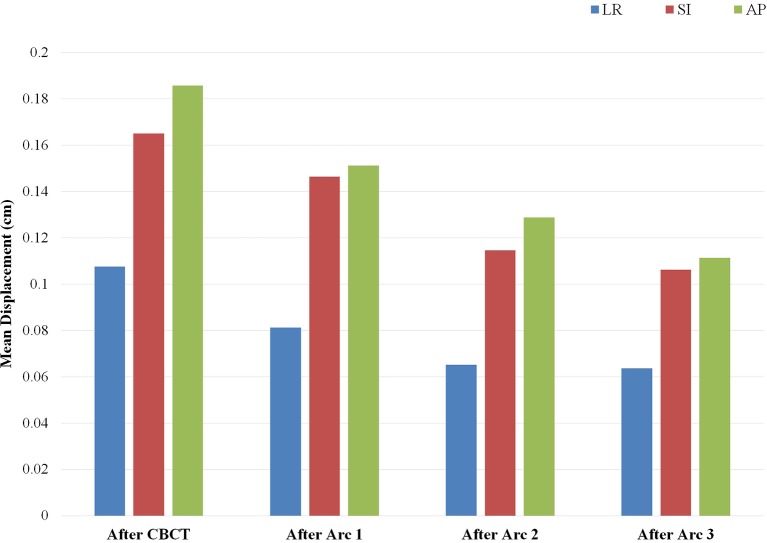
Mean implemented shifts for prostate displacement sequentially over the phases of treatment: from cone beam computed tomography (CBCT) to planar imaging just prior to initiation of arc 1, and between each of the partial arcs incorporating the displacement detected on the image pair obtained subsequent to the corresponding arc. Depicted mean shifts are relative to the immediate predecessor intra-fractional displacement and are based on an institutional threshold of implementing shifts with displacement ≥1 mm. Arc 4 is not included, as there is no IGRT performed after completion of the fourth arc delivery.

The patterns of prostate displacement longitudinally across the phases of treatment delivery are displayed in Figure 3, which displays the mean shifts implemented for each intra-fractional phase relative to the immediate preceding intra-fractional shifts. Mean displacement was most pronounced during the initial period from CBCT to planar imaging prior to beam-on of the first arc, and decreased with each of the subsequent arcs.

### Severe Toxicity

Overall, six patients experienced a grade ≥3 GI or GU toxicity. Mean prostate displacement in patients with and without grade ≥3 GI or GU toxicities are presented in Table 3. No significant differences were found between mean inter- or intra-fractional displacement in any single direction and development of grade ≥3 toxicity (inter-fractional *p* = 0.99, 0.98, and 0.46 and intra-fractional *p* = 0.80, 0.84, and 0.10 in the LR, SI, and AP directions, respectively) (Table 3).

**Table 3:** Mean inter- and intra-fractional prostate displacement (in cm) for patients with vs. without development of severe toxicity.

	**Inter-fractional (cm)**			**Intra-fractional (cm)**		
	**LR**	**SI**	**AP**	**LR**	**SI**	**AP**
**No Grade 3 toxicity**	−0.03 ± 0.30	−0.06 ± 0.43	−0.06 ± 0.45	0.07 ± 0.05	0.13 ± 0.07	0.14 ± 0.09
**Grade 3 toxicity**	0.02 ± 0.16	0.03 ± 0.37	0.07 ± 0.41	0.07 ± 0.03	0.14 ± 0.07	0.08 ± 0.06
***p*****-value**	0.99	0.98	0.46	0.80	0.84	0.10

## Discussion

To our knowledge, the present study is the largest analysis of prostate displacement and required margin calculation to date. We found that in the absence of intra-fractional corrections, minimum SI and AP margins of 2.7 and 3.1 mm were required, with 2.0 and 5.4% of patients having mean intra-fractional SI and AP displacements of > 3 mm, respectively. As the current data pertain to patients treated on a gantry-mounted linear accelerator with implanted fiducial markers but without specific rectal immobilization devices or tracking devices, the suggested margins can be readily extrapolated to other clinical settings in which such devices might not be available. Inter-fractional variations were of a much greater magnitude, with minimum recommended SI and AP margins of 1.52 and 1.45 cm, respectively. As a result of the greater standard deviation in mean shift between fractions, calculated margins for inter-fractional displacement were quite large. These confirm that inter-fractional motion management is required for SBRT, as margins of this magnitude would be prohibitive of high dose-per-fraction treatments.

On examination of prostate displacement over the sequential phases of treatment, including initial IGRT and each partial arc, we identified that the greatest displacement in all three cardinal directions occurred in the period between CBCT and the repeat planar imaging obtained just before initiation of beam-on. Furthermore, the treatment preparation time required for inter-fractional alignment accounted for approximately half of the overall treatment time. This finding reinforces the importance of obtaining repeat orthogonal imaging after anatomic verification prior to initiating the first arc delivery, which would considerably reduce the margins required for intra-fractional motion. Displacement over the course of the arcs did not increase, and actually decreased slightly in a longitudinal fashion. One should note that the correction threshold of 1 mm used in our IGRT protocol may also lead to overestimation of the intra-fractional motion due to potential residual sub-mm error carried over to subsequent arcs.

Our measured intra-fractional displacements and calculated margins are similar to previously published data for SBRT and conventional fractionation (8, 28–32). Mean displacement was greatest in the SI and AP directions, and was of similar magnitude in these two directions though slightly more pronounced anteriorly-posteriorly, which is consistent with prior reports (8, 16, 23, 25). Greater intra-fractional displacement has also been reported, up to 2.0 ± 1.8 mm (SI) and 1.9 ± 2.0 mm (AP) based on pre- and post-treatment orthogonal imaging (19). Larger required margins have been reported as well, up to 4.4 mm SI and 5.2 mm AP based on pre- and post-treatment imaging during SBRT (25), and 5.4 mm (SI) and 5.0 mm (AP) based on Calypso data during conventional fractionation (27). Notably, some studies have noted translations as small as 0.01 ± 0.23 mm (LR), 0.11 ± 0.64 mm (AP), and 0.21 mm ± 0.12 (SI) with incorporation of a rectal distension device and real-time kV infraction monitoring (20). A strength of the present study, however, is the large patient cohort, as the majority of prior published studies have included fewer than 100 patients, and many with fewer than 50 patients. Our large patient numbers provide weight to our conclusions regarding average target displacement and required margins. Indeed, our standard deviations of 0.5–0.9 mm for prostate displacement are smaller than those reported in many published datasets, including those which found smaller mean directional displacements, as standard deviations of up to 2 mm have been reported (8, 9, 16, 19, 25).

Importantly, the calculated margins reflect a scenario in which intra-fractional shifts are not applied in real-time. With incorporation of real-time intra-fractional motion monitoring, it is conceivable that smaller margins might be practical and feasible, although systematic errors related to target delineation or geometric uncertainty in the image guidance system would still remain. Conversely, in the absence of real-time intra-fractional monitoring and corrections, the commonly used 3 mm posterior margin may be insufficient for adequate target coverage. Indeed, measurements from real-time electromagnetic beacon data have shown that in the absence of intra-fractional corrections, a posterior margin of 3 mm would result in unacceptably low (93%) CTV coverage (32). If we assume 1–2 mm margins are necessary for systematic errors related to contouring and machine performance (33–35), then the minimum PTV margins required in the absence of frequent intra-fractional monitoring and correction would be 2.9–3.9 mm LR, 3.7–4.7 mm SI, and 4.1–5.1 mm AP. The fact that a greater posterior margin is necessary is practically relevant, as many contemporary SBRT protocols allow for smaller posterior margins (5, 36–39). For instance, in the landmark PACE-B trial, margins were 4–5 mm non-posteriorly and 3–5 mm posteriorly (5). The protocol mandated intra-fractional monitoring for patients receiving SBRT on a gantry-mounted linear accelerator who had an anticipated treatment time of >3 min; however, this appeared to account for a very small minority (~0.7%) of patients receiving SBRT on the trial. Thus, among patients receiving gantry-mounted linear accelerator-based SBRT, most were treated in under 3 min, and therefore no intra-fractional motion correction was used. In general, the margins used on the trial seem to be adequate, though even for short treatment times, our data suggest that a total posterior margin of 3 mm may be too narrow, given residual errors that must be accounted for in the PTV, and a minimum margin of 4 mm may be more reasonable in the absence of any intra-fractional motion management.

With respect to severe toxicity, though grade ≥3 toxicities were rare, we evaluated whether these events may have been due to large prostate displacements or variations. We did not find an association between the magnitude of mean prostate displacement and development of grade ≥3 GI or GU toxicity. This finding suggests that initial treatment planning dosimetry and intrinsic patient factors may be most responsible for these severe adverse events (40–42). In contrast, Choi *et al*. assessed the relationship between grade ≥2 toxicity and intra-fractional prostate displacement during CyberKnife SBRT, and found that both GI and GU grade ≥2 toxicity were associated with the degree of AP displacement (26). The association between lower grade toxicity (1 and 2) and shift data is an area for future analysis with our large dataset.

There are several limitations to the present study. First, as we have used intra-fractional orthogonal imaging (in the present study, 3 times between partial arcs) to determine intra-fractional prostate motion, our acquired imaging is, by definition, both intermittent and instantaneous rather than continuous. This method creates potential for several uncertainties, including whether the captured position represents a sustained vs. transient displacement, and whether the maximum magnitude of displacement has been missed between imaging sets. Incomplete capturing of larger displacements could result in underestimation of required margins. However, as our recorded mean displacements were similar to—if not slightly larger than—many studies using continuous or more frequent monitoring (such as CyberKnife, Calypso Beacon transponders, cine-MRI, and real-time ultrasound studies) (20, 24, 26, 43, 44), this may not be the case. The lack of rotational data is another potential study limitation; however, data from the recent phase II TROG 15.01 SPARK trial of continuous kV intra-fraction monitoring suggest that rotational changes appear to fall within PTV margins used to account for translational motion, and the authors conclude that CTV-to-PTV expansions should focus on accounting for translational displacements (45).

In summary, we propose required PTV margins for prostate SBRT based on a large cohort of over 200 patients treated using intra-fractional kilovoltage imaging. Our findings fall within the mid-range of required margins and prostate translations that have been published, and are generally encompassed by the commonly used expansions for SBRT. In this context, documented prostate displacement does not appear to be correlated with development of late severe toxicity. Our results further support the importance of image-guided setup and intra-fractional motion monitoring if margins on the order of 3–5 mm are to be employed, particularly with regard to implementing a reduced, anisotropic posterior margin.

Our results are thus applicable to prostate SBRT delivery without real-time monitoring or immobilization of neighboring structures such as the rectum. Since the proposed margin calculations do not take into account these motion management strategies, utilization of technologies incorporating more frequent intra-fractional corrections may allow for even narrower margins. Such technologies include electromagnetic transponders and MR-LINACs, the latter of which will also allow for online adaptation based on the deformation of adjacent organs at risk.

## Data Availability Statement

The datasets for this article are not publicly available because of institutional policies regarding protection of the data. Requests to access the datasets should be directed to Amar U. Kishan, aukishan@mednet.ucla.edu.

## Ethics Statement

The studies involving human participants were reviewed and approved by UCLA institutional review board. Written informed consent for participation was not required for this study in accordance with the national legislation and the institutional requirements.

## Author Contributions

RL-E, DR, LV, NN, MC, and AK contributed to authoring the manuscript. GQ-G, JJ, ZS, DR, LV, MS, PK, CK, AK, and MC contributed to data collection and treatment planning.

### Conflict of Interest

The authors declare that the research was conducted in the absence of any commercial or financial relationships that could be construed as a potential conflict of interest. The handling Editor declared a past co-authorship with one of the authors AK.
